# Low-Temperature Biodegradation of Lignin-Derived Aromatic Model Monomers by the Cold-Adapted Yeast *Rhodosporidiobolus* *colostri* Isolated from Alpine Forest Soil

**DOI:** 10.3390/microorganisms10030515

**Published:** 2022-02-26

**Authors:** Rosa Margesin, Thomas Marek Ludwikowski, Andrea Kutzner, Andreas Otto Wagner

**Affiliations:** Department of Microbiology, Universität Innsbruck, Technikerstraße 25, 6020 Innsbruck, Austria; thomas.ludwikowski@student.uibk.ac.at (T.M.L.); andrea.kutzner@student.uibk.ac.at (A.K.); andreas.wagner@uibk.ac.at (A.O.W.)

**Keywords:** yeast, *Rhodosporidiobolus colostri*, cold-adapted, biodegradation, lignin-derived aromatic monomers, *p*-coumaric acid, 4-hydroxybenzoic acid, ferulic acid, vanillic acid

## Abstract

The contribution of cold-adapted yeasts to the emerging field of lignin biovalorization has not yet been studied. The red-pigmented basidiomycetous yeast strain *Rhodosporidiobolus* *colostri* DBVPG 10655 was examined for its potential to degrade five selected lignin-derived aromatic monomers (syringic acid, *p*-coumaric acid, 4-hydroxybenzoic acid, ferulic acid, and vanillic acid). The strain utilized *p*-coumaric acid, 4-hydroxybenzoic acid, and ferulic acid not only as the sole carbon source; full biodegradation occurred also in mixtures of multiple monomers. Vanillic acid was not utilized as the sole carbon source, but was degraded in the presence of *p*-coumaric acid, 4-hydroxybenzoic acid, and ferulic acid. Syringic acid was utilized neither as the sole carbon source nor in mixtures of compounds. Biodegradation of lignin-derived aromatic monomers was detected over a broad temperature range (1–25 °C), which is of ecological significance and of biotechnological relevance.

## 1. Introduction

Lignin is one of the most abundant and energy-dense polymers on earth. It can be found in the secondary cell wall of lignocellulosic plants where it provides structural strength, impermeability, water transport in the cell wall, and protection from plant pathogens [1]. Due to its complex structure, lignin is one of the most recalcitrant polymers [2,3,4]. The complex aromatic heteropolymer is a rich resource of renewable aromatic compounds that could be used for a wide range of biotechnological applications [1,5]. The potential of microorganisms for the biochemical conversion of lignin into value-added products and renewable fuels such as biogas and bioethanol has been demonstrated [6,7,8].

Microbial breakdown of lignin has been attributed primarily to basidiomycetous wood rotting fungi [2,7,9]. More recently, the ligninolytic potential of bacteria has been recognized [2,10,11]. Information on the interaction of yeasts, unicellular eukaryotes, with lignin is scarce [3]. Yeasts are not known as primary degraders of complex recalcitrant polymers such as the macromolecule lignin [3,9,12]. However, yeasts contribute to lignin depolymerization by utilizing the aromatic compounds produced after the delignification by filamentous fungi, thereby acting as detoxification agents [9]. The first step of lignin biodegradation results in the release of aromatic compounds, which are toxic to many microorganisms already at low concentrations. To eliminate toxicity, the aromatic compounds have to be degraded or converted to less toxic or nontoxic compounds by cleavage of the aromatic ring; the resulting compounds can be used as a carbon source [5].

The assimilation of intermediates of lignin degradation, such as various low-molecular-weight aromatic compounds (e.g., ferulic acid, 4-hydroxybenzoic acid, *p*-coumaric acid, vanillic acid, and protocatechuic acid) by basidiomycetous and ascomycetous yeast strains has been reported in a number of studies [9,13,14,15,16,17,18,19,20,21]. Basidiomycetous yeasts have a greater capacity to utilize low-molecular-mass lignin-derived aromatic compounds as sole sources of carbon and energy than ascomycetous yeasts [9].

The degradation of lignin-derived aromatic compounds by yeasts has been generally assessed under mesophilic temperature conditions (mostly at 24–30 °C; [3,9,16,18,19,21,22]); Sampaio [17] applied temperatures in the range of 17–22 °C. In contrast to the recently shown bacterial utilization of lignin and linin-related compounds at low and moderate temperatures [11,23,24], knowledge on the role of degradation capacities of yeasts in these temperature regimes is still scarce. Understanding the contribution of yeasts to lignin degradation in low temperature areas is of high ecological significance. Cold-adapted microorganisms, including yeasts, play a key role in their natural habitats for many processes, such as nutrient cycling and litter degradation [25,26]. In addition, microbial activity at low temperatures offers a wide range of advantages for biotechnological processes and is of particular interest for low-energy treatments [27].

It was the aim of this study to assess the capability of the culturable cold-adapted yeast strain *Rhodosporidiobolus colostri* DBVPG 10655, isolated from soil from an Alpine deciduous forest site [28], to degrade representative aromatic model compounds associated with lignin degradation. The five lignin-derived aromatic monomers studied were syringic acid, *p*-coumaric acid, 4-hydroxybenzoic acid, ferulic acid, and vanillic acid. Biodegradation of these compounds was studied, both individually and in mixtures (containing multiple compounds), over the growth temperature range of the studied yeast strain (1–25 °C). Here, we report for the first time the degradation potential for lignin model compounds by a cold-adapted basidiomycetous yeast.

## 2. Materials and Methods

### 2.1. Strain

The yeast strain used in this study was isolated from soil from an Alpine deciduous forest site located 8 km south of Bozen/Bolzano on a small peak, Kleiner Priol, at an altitude of 545–570 m above sea level and identified as *Rhodosporidiobolus colostri* (*T. Castelli*) Q.M. Wang, F.Y. Bai, M. Groenew, and Boekhout [29] as described in [28]. The strain was deposited in the Industrial Yeast Collection DBVPG under the number DBVPG 10655. The strain was stored at −80 °C using ROTI©Store yeast cryovials (CarlRoth, Karlsruhe, Germany).

### 2.2. Chemicals

Chemicals syringic acid (SA; Alfa Aesar 5003), *p*-coumaric acid (CA; Sigma C9008), 4-hydroxybenzoic acid (HBA; Serva 25271), protocatechuic acid (PCA; Sigma-Aldrich 375880, as possible intermediate), *trans*-ferulic acid (FA; Sigma-Aldrich 128708), vanillin (Merck 818718, as possible intermediate), and vanillic acid (VA; Merck 841025) were of chromatographic pure grade. Stock solutions of tested chemicals (0.5–1 M) were prepared in DMSO and stored at 4 °C. Preliminary studies showed that the final amount of DMSO did not affect yeast growth.

### 2.3. Biodegradation of Lignin-Derived Aromatic Monomers

The biodegradation assays were carried out at 10 °C and 150 rpm in 100 mL Erlenmeyer flasks with screw caps containing 20 mL of mineral salts medium (MM; [11]) supplemented with a trace element and a vitamin solution [11] and the target compound(s) as sole carbon source(s). The pH of the medium was adjusted to 7.0 after the addition of the compound(s).

For inoculation, a preculture prepared in Reasoner’s 2A (R2A) broth was prepared. The pH-neutral medium was composed of yeast extract (0.5 g/L), glucose (0.5 g/L), starch (0.5 g/L), Bacto Tryptone (0.5 g/L), sodium pyruvate (0.3 g/L), K_2_HPO_4_ (0.3 g/L), and MgSO_4_·7H_2_O (0.05 g/L). The yeast cells were separated by centrifugation (10,000× *g* for 10 min), washed twice with sterile MM, and suspended in MM. The initial (t_0_) optical density at 600 nm (OD_600_) in the flasks was adjusted to 0.05 by inoculation with an aliquot of *R. colostri* preculture. To ensure sufficient aeration, culture flasks were opened regularly under sterile conditions. Two negative controls contained (i) sterile medium supplemented with the target compounds and (ii) inoculated medium without the target compounds.

Growth (OD_600_), pH, and the concentration of the target compounds were monitored in samples collected at regular time intervals.

**Figure 1:**
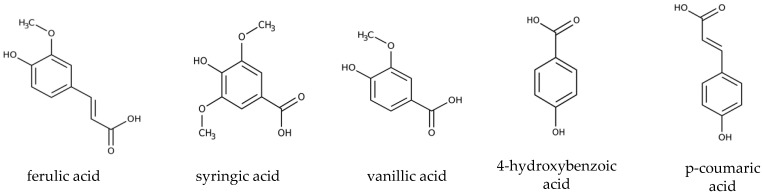
Chemical structure of model compounds used in the present study. Structures were created using http://www.strukturformelzeichner.de/.

To study the biodegradation of lignin-derived aromatic monomers at 10 °C, the medium was supplemented with the target compounds (SA, CA, HBA, FA, VA; Figure 1), either individually (5 mM, final concentration) or in a mixture (2.5 and 5 mM final concentration of each compound). The biodegradation of mixtures of lignin-derived aromatic monomers was additionally assessed in mixtures without SA (four compounds: CA, HBA, FA, VA; each 5 mM) and without SA and VA (three compounds: CA, HBA, FA; each 5 mM).

The effect of temperature on the biodegradation of 5 mM FA and of a mixture of FA, CA, HBA, and VA (each 5 mM) was determined at temperatures ranging from 1 to 30 °C (5 °C intervals).

The toxicity (growth-inhibiting effect) of VA was studied at 10 °C and 20 °C in R2A broth supplemented with VA at concentrations of 0.625, 1.25, 2.5, and 5 mM. The control was prepared in R2A without VA.

### 2.4. HPLC Analysis

Lignin-derived aromatic monomers were quantified via HPLC analysis as described previously [11]. Prior to HPLC analysis, samples were centrifuged at 20,000 × *g* for 10 min, and supernatants were frozen at −20 °C. The analysis was performed on a Shimadzu Prominence system equipped with a RFQ Fast Acid column (50 × 7.8 mm, Phenomenex, Aschaffenburg, Germany) as described previously [30]. Detection was carried out via a UV detector at 220 nm and crosschecked at 270 nm. As external standards, SA, CA, HBA, PCA, FA, VA, and vanillin were used in concentrations of 1, 5, and 10 mM.

## 3. Results

No abiotic losses of lignin-derived aromatic monomers, whether individually or in mixture, were detected in any of the biodegradation experiments. Thus, the decrease in the monomer concentrations in the inoculated flasks could be attributed to biodegradation. The pH-neutral conditions in the culture media were maintained during growth and biodegradation.

### 3.1. Biodegradation of Individual Lignin-Derived Aromatic Monomers

**Figure 2:**
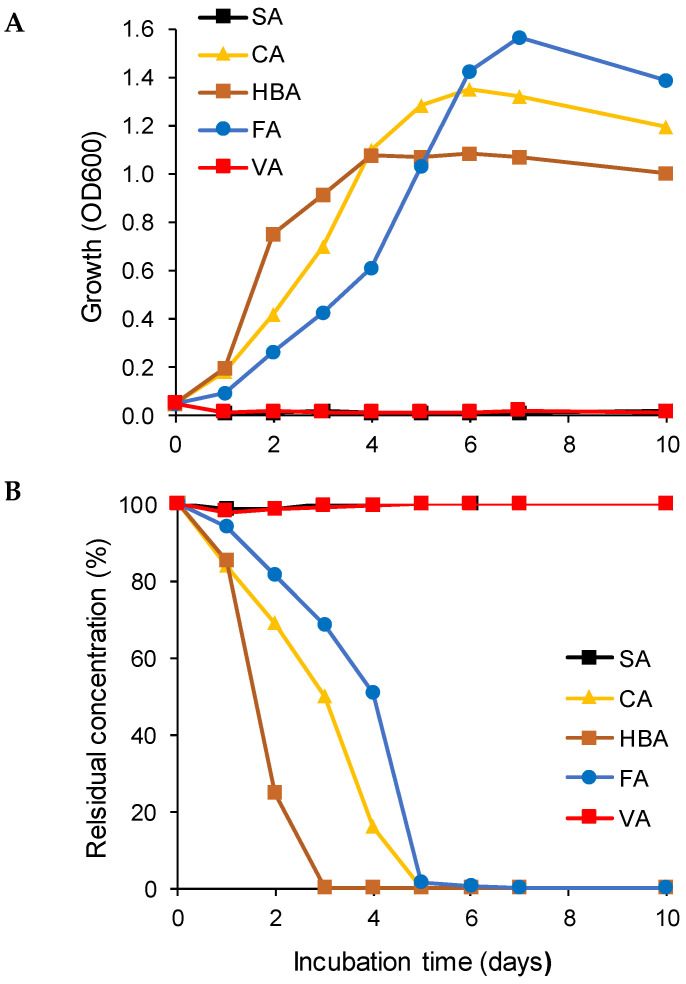
Utilization as the sole carbon source ((**A**): growth; and (**B**): degradation) of lignin-derived aromatic monomers (SA, syringic acid; CA, *p*-coumaric acid; HBA, 4-hydroxybenzoic acid; FA, *trans*-ferulic acid; VA, vanillic acid; 100% = 5 mM) by *R. colostri* DBVPG 10655 at 10 °C (mean values of three replicates; SDs ≤ 10%).

The studied strain *R. colostri* DBVPG 10655 was able to utilize three out of the five tested lignin-derived aromatic monomers (5 mM CA, HBA, FA), if supplemented individually as the sole carbon and energy source at 10 °C; SA and VA could not be utilized (Figure 2). Biodegradation paralleled growth (OD_600_). Biomass production was highest during the biodegradation of FA and was lowest from HBA degradation. However, OD_600_ was not indicative of the degradation capacity. Among the degraded compounds, HBA was preferentially utilized and fully degraded after 3 days of incubation, followed by CA and FA (Figure 2).

### 3.2. Biodegradation of LigninDerived Aromatic Monomers in Mixture

**Figure 3:**
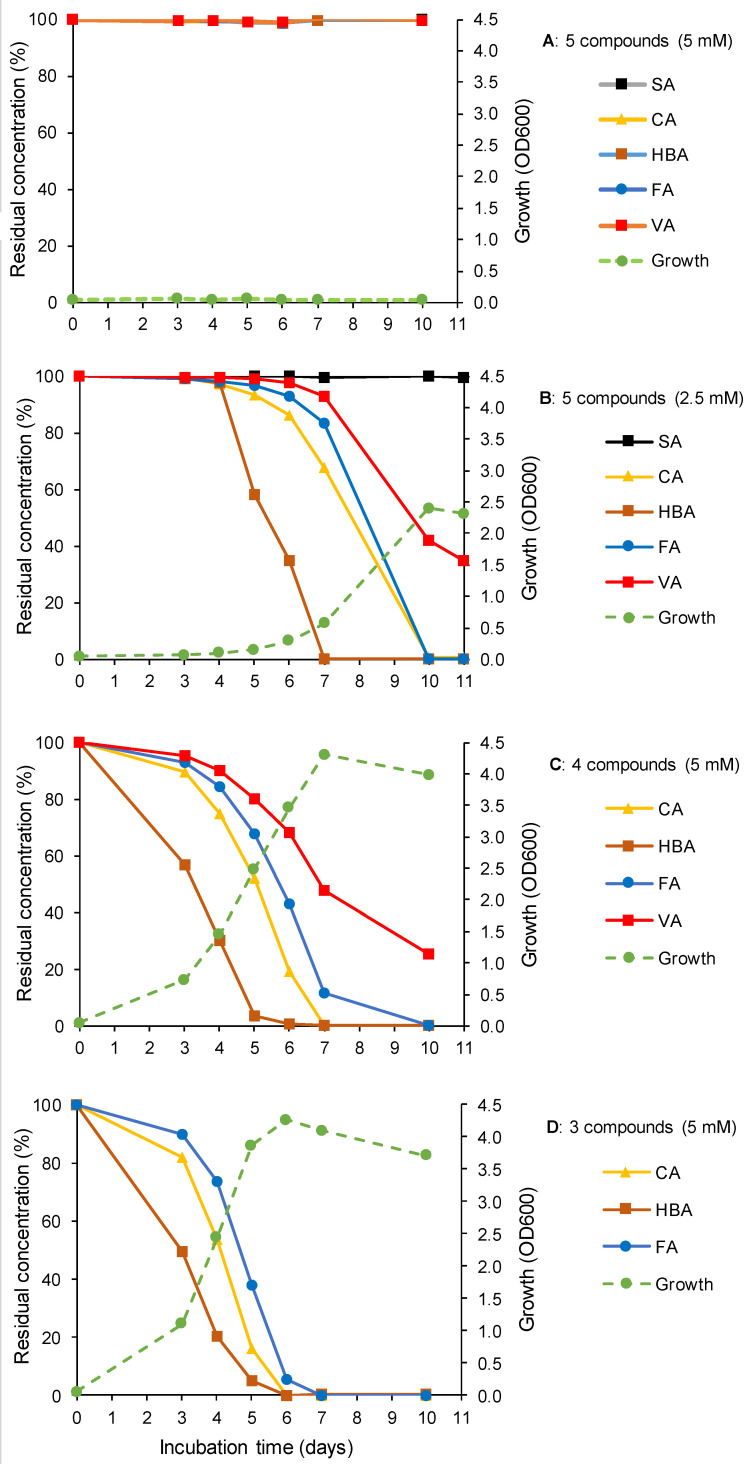
Biodegradation performance and growth of *R. colostri* DBVPG 10655 at 10 °C in the presence of five ((**A**) 100% = 5 mM; (**B**) 100% = 2.5 mM), four ((**C**) 100% = 5 mM), and three ((**D**) 100% = 5 mM)) lignin-derived aromatic monomers (SA, syringic acid; CA, *p*-coumaric acid; HBA, 4-hydroxybenzoic acid; FA, *trans*-ferulic acid; VA, vanillic acid) as sole carbon sources (mean values of three replicates; SDs ≤ 10–15%).

Biodegradation of the five lignin-derived aromatic monomers by *R. colostri* DBVPG 10655 was also evaluated in a mixture of these compounds at 10 °C. The mixture of all five tested compounds (SA, CA, HBA, FA, VA) did not support growth and, thus, biodegradation at a concentration of 5 mM of each of the compounds (Figure 3A). However, a concentration of 2.5 mM of each of the five compounds resulted not only in growth and biodegradation of those three compounds (CA, HBA, FA) that were also degraded if supplemented individually (CA, HBA, FA; Figure 2); VA was additionally degraded (Figure 3B). The preferential utilization of HBA over CA and FA reflected the results obtained when the utilization of the tested monomers was evaluated individually.

To find out whether one of the five lignin-derived aromatic monomers inhibited biodegradation in the mixture of these compounds at a concentration of 5 mM, the effect of mixtures of four (CA, HBA, FA, VA; without SA) and three (CA, HBA, FA; without SA and VA) lignin-derived aromatic monomers (each 5 mM) on growth and biodegradation was assessed. Indeed, the omission of SA in the four compounds mixture resulted in the biodegradation of all four monomers, including VA (HBA > CA> > FA > VA) (Figure 3C). Furthermore, the three-compound mixture demonstrated the ability of the strain to degrade HBA, CA, and FA at 10 °C (Figure 3D).

An increase in the number of compounds in the mixtures resulted in delayed growth and biodegradation (Figure 3B–D). A considerable lag phase for growth was noted in the mixture of five compounds (2.5 mM) compared to growth in the presence of three and four compounds (5 mM).

While 5 mM HBA was almost fully degraded after 5 days at 10 °C in the mixtures of three and four compounds, seven days were needed for full degradation of 2.5 mM HBA in the five-compound mixture. Of 5 mM FA, around 90% or more had disappeared after 6 and 7 days in the three- and four-compound mixtures, respectively, while 83% of 2.5 mM FA was still detectable in the five-compound mixture after 7 days. Similar results were observed for CA. More than 50% of 5 mM VA was degraded within 7 days in the four-compound mixture, while three more days were needed for the same degradation performance of 2.5 mM VA in the five-compound mixture. However, full VA degradation was obtained within the considered incubation time.

### 3.3. Effect of Temperature on Growth and Biodegradation

**Figure 4:**
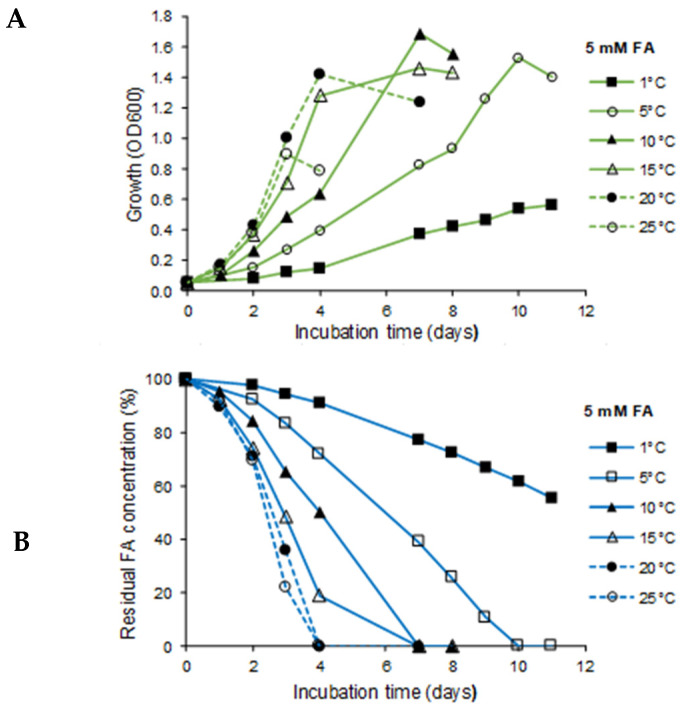

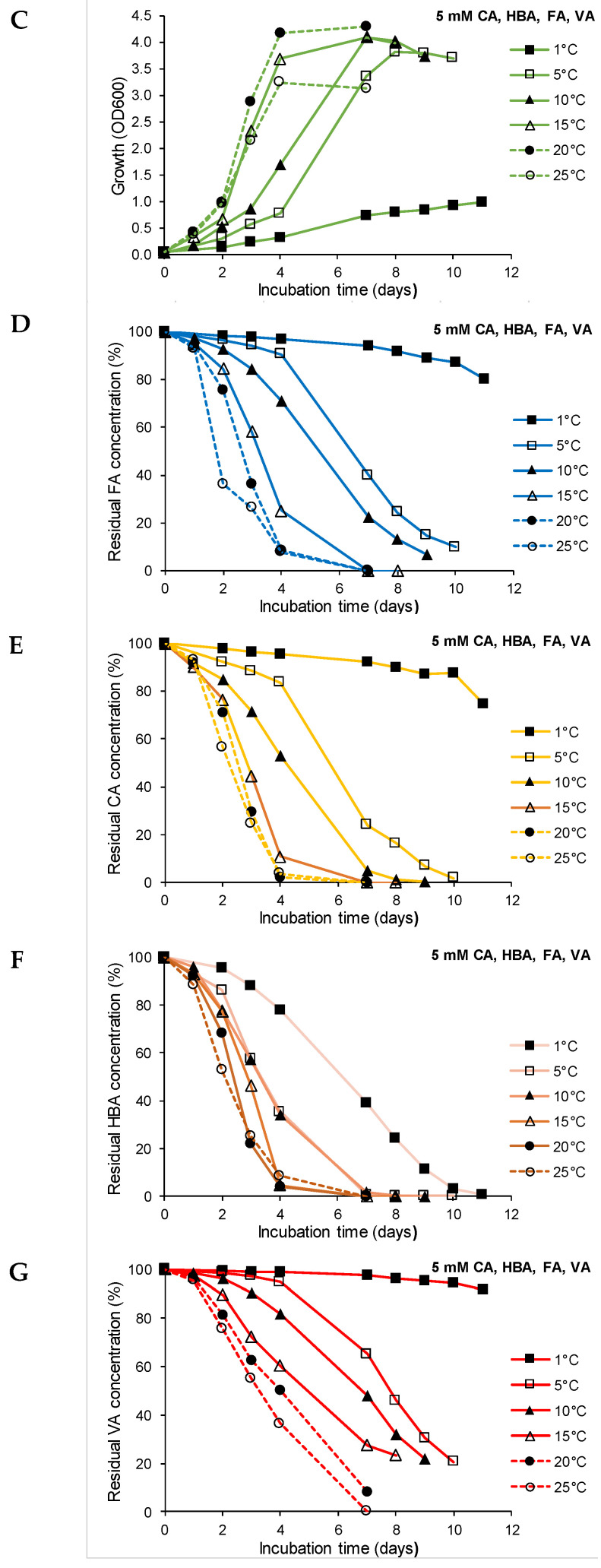
Effect of temperature on growth (**A**) and degradation (**B**) of 5 mM FA (100% = 5 mM; lower panel) by *R. colostri* DBVPG 10655. Effect of temperature on growth (**C**) and degradation (**D**–**G**) of a mixture of FA, CA, HBA, and VA (100% = 5 mM) by *R. colostri* DBVPG 10655. FA, *trans*-ferulic acid; CA, *p*-coumaric acid; HBA, 4-hydroxybenzoic acid; VA, vanillic acid (mean values of three replicates; SDs ≤ 10–15%).

The investigation on the effect of temperature on the biodegradation of FA (5 mM) as a single lignin-derived aromatic monomer and of the mixture of the four monomers CA, HBA, FA, and VA (each 5 mM) on growth and biodegradation demonstrated the degradative activity of *R. colostri* DBVPG 10655 over a broad temperature range (1–25 °C). No growth occurred at 30 °C. The same growth temperature range was observed in complex media. A higher growth temperature led to faster biodegradation. However, the order of degradation preference (HBA > CA > FA > VA) was temperature-independent (Figure 4).

The highest biomass production was observed in the temperature range 5–20 °C, in the presence of FA both as a single monomer and in the mixture of four compounds. Biomass production at 25 °C was significantly lower, albeit still significantly higher than at 1 °C (Figure 4). Since all four compounds present in the mixture could be degraded, biomass production was significantly higher in the mixture (more than twofold) than with FA as sole carbon source.

Full biodegradation of 5 mM FA was obtained at 5–25 °C; its utilization at 1 °C was significantly higher when provided as sole carbon source compared to the mixture (Figure 4). CA and VA were also fully degraded in the mixture at 5–25 °C; however, almost no degradation occurred at 1 °C within the tested incubation temperature (Figure 4). Interestingly, this was not the case for HBA, which was the only monomer being effectively degraded over the whole temperature range tested, i.e., also at 1 °C.

### 3.4. Toxicity of VA

**Figure 5:**
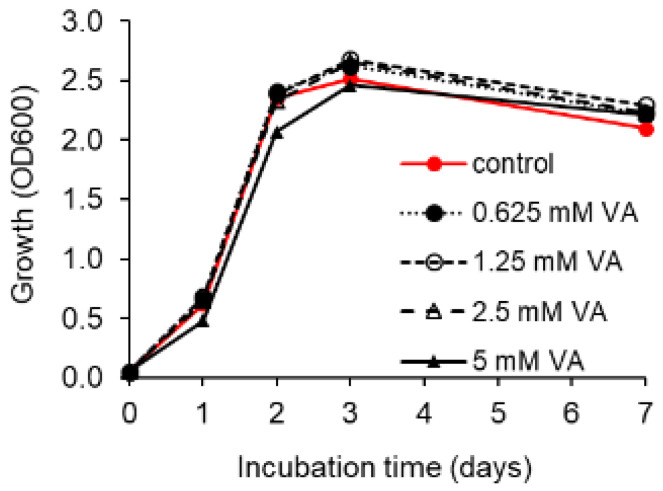
Effect of various concentrations of VA (vanillic acid) on growth (OD_600_) of *R. colostri* DBVPG 10655 in complex medium at 10 °C (control = growth in R2A broth without VA) (mean values of three replicates; SDs ≤ 10%).

Studies on the toxicity of VA demonstrated that its presence in complex medium had no negative effect on the growth of *R. colostri* DBVPG 10655. Growth was slightly delayed in the presence of 5 mM VA. However, the biomass obtained in the stationary growth phase was not significantly influenced by the presence of the VA concentrations tested (0.625–5 mM) (Figure 5). The same result was observed at 20 °C. Thus, the inability of the strain to degrade VA as the sole carbon source could not be attributed to toxicity issues.

## 4. Discussion

Here, we described the degradation of lignin-derived aromatic monomers by the culturable red-pigmented basidiomycetous yeast *R. colostri* DBVPG 10655 (basionym *Mycotorula colostri T. Castelli*) which was isolated from soil from an Alpine deciduous forest site. The capability of culturable bacterial strains from this forest site to degrade a range of organic compounds, including lignin and lignin-related aromatic compounds, at low and moderate temperatures has been previously described [24]. Cold-adapted yeast strains were previously isolated from this site [28]; however, their degradation potential has not yet been investigated.

Representatives of the genus *Rhodosporidiobolus* have been isolated from various sources, including cold ecosystems [31,32], apple and pear surfaces [33], apple pulps [34], grapes [35], marine ecosystems associated with invertebrates such as sponges [36] and corals [37], and waste deposit of a leaf-cutting ant [38]. The frequent association of members of the genus *Rhodosporidiobolus* with plant structures points to their ability to utilize lignin-derived compounds.

Data obtained in this study demonstrate the capability of the investigated yeast strain *R. colostri* DBVPG 10655 to utilize three lignin-derived aromatic monomers (CA, HBA, FA) over a broad temperature range (1–25 °C), independently of whether they were present as individual compounds or in a mixture. The degradation of CA, HBA, and FA indicates the utilization of two main branches of the upper funneling catabolic pathways for the metabolization of lignin-derived compounds [10]: the *p*-coumaryl branch (utilization of CA and HBA) and the coniferyl branch (utilization of FA). The preferential degradation of compounds of the *p*-coumaryl branch compared to the coniferyl branch (HBA > CA > FA) was clearly visible. This has also been reported for bacterial strains [11,39] and might be attributed to the absence of a methyl group in these structures of the *p*-coumaryl branch.

In our study, the lignin-derived aromatic monomer VA was not degraded if present as the sole carbon source. Interestingly, *R. colostri* DBVPG 10655 was able to utilize VA in the presence of CA, HBA, and FA at temperatures ranging from 5 to 25 °C. VA belongs, like FA, to the coniferyl branch. We hypothesize that the clearly visible, preferential utilization of FA compared to VA could be an indication of the expression of enzymes during FA degradation, which might induce further enzymes needed for the metabolization of degradation of VA and, thus, allow its degradation. It could also be possible that the strain downregulated the gene necessary for the expression of the involved enzyme(s), i.e., these enzymes are not expressed if VA is the sole carbon source. However, a study to elucidate gene expression is planned, and we hope to clarify regulation mechanism via this approach. Since our data clearly demonstrate that VA had no growth-inhibiting effect on the strain at the tested concentration (5 mM) (growth was only marginally delayed), a toxic effect of VA could be excluded.

Several pathways have been described for the microbial degradation of FA. The conversion of FA to VA has been observed in oleaginous yeasts, such as *Cryptococcus curvatus*, *Rhodosporium toruloides*, and *Trichosporon guehoe* [5]. The degradation of VA by fungi is poorly studied [40]. In the present study, vanillin was not detectable at any stage of any of the biodegradation experiments. The absence of this metabolite in yeasts has previously been observed [22]. The production of vanillin from VA is known from bacteria and filamentous fungi, while reports on vanillin-producing yeasts are rare [5], which is probably related to its toxic effect [41]. Vanillin is the product of VA reduction in fungi [42,43], while it is the oxidation precursor to VA in bacteria [44].

VA is converted by bacteria, yeasts, and filamentous fungi to three main compounds (protocatechuic acid, catechol, and hydroxyquinol), after which the ring is cleaved and converted before entering the TCA cycle [5]. In our study, we observed PCA in the VA-containing mixture only temporarily and in very low amounts (0.5–0.6 mM). This points to the production of PCA from VA; however, further studies are needed to confirm this hypothesis. A study on the assimilation of lignin-related low-molecular-weight aromatic compounds by 332 heterobasidiomycetous yeasts demonstrated that this group of yeasts is better adapted to utilize simple aromatic compounds via the PCA branch than via the catechol branch of the 3-oxoadipate pathway [17]. In this study, ring cleavage of catechol was not investigated, since, in preliminary tests, the studied strain did not produce catechol-1,2 dioxygenase or catechol-2,3-dioxygenase, which are needed for catechol degradation [5].

The inability of *R. colostri* DBVPG 10655 to utilize SA as the sole carbon source, whether supplemented individually or present in a mixture of lignin-derived aromatic monomers, indicates the absence of the metabolization ability of lignin-derived compounds of the sinapyl branch by the studied yeast strain. This could be the result of stearic hindrance or of toxicity due to the presence of methoxy groups [45]. In our study, the absence of growth and biodegradation of HBA, CA, FA, and VA in the presence of 5 mM SA, but the ability to degrade these compounds in the presence of 2.5 mM SA, points to the toxic effect of SA. The inability of heterobasidiomycetous yeasts to assimilate SA has previously been reported [17].

A number of yeasts, some of them isolated from soil, have been described to utilize a range of lignin-derived aromatic monomers, such as FA, HBA, CA, PCA, and VA, as sole carbon and energy sources [13,14,15,16,17,18,19,20,21]. The biodegradation of mixtures of lignin-derived model compounds, as described in our study, has only been reported for bacteria [39]. Gu et al. [46] observed a synergistic inhibitory effect of the presence of multiple lignin-derived compounds on lignocellulosic ethanol fermentation by *Saccharomyces cerevisiae*; the inhibition intensity order was VA > SA > HBA [46]. The inhibition was attributed to the combined effect of the damage of the cytoplasmic membrane and the intracellular acidification due to the presence of these compounds. Growth of the yeast *Rhodosporidium toruloides* in lignocellulosic biomass hydrolysates was severely inhibited by the presence of compounds such as FA, VA, and vanillin [47].

An important factor in biotechnological applications is temperature. The degradation of lignin-derived aromatic monomers by yeasts has been generally assessed under mesophilic temperature conditions (mostly at 24–30 °C; [3,9,16,18,19,21,22]). Sampaio [17] applied temperatures in the range of 17–22 °C. To the best of our knowledge, the low-temperature biodegradation of lignin-derived aromatic monomers by yeasts has not yet been described. We could demonstrate that *R. colostri* DBVPG 10655 is an efficient degrader of 5 mM CA, FA, and VA at temperatures ranging from 5 °C to 25 °C within 10 days. HBA was the only of the studied monomers degraded efficiently over the whole temperature range tested (1–25 °C), i.e., also at 1 °C. This could be caused by the fact that this compound was preferentially degraded and, thus, biodegradation was extended over the whole growth temperature range.

Overall, the broad-temperature degradation performance of the studied cold-adapted yeast strain is an advantage in environments undergoing temperature fluctuations. The ability of cold-adapted yeasts to degrade aromatic hydrocarbons at low temperatures has previously been reported [25,48]. Knowledge on the microbial degradation of lignin-derived aromatic compounds (with great industrial and commercial potential) is of biotechnological interest. In addition, the fate of aromatic compounds produced and released to the environment, particularly those related to human activities, is also of great interest for bioremediation strategies, such as wastewater treatment [9,19] or biogas processes [49]. Lastly, the role of cold-adapted yeasts in lignin degradation in nature is of ecological interest. The adaptation of microorganisms to cold temperatures is based on a wide and complex range of adjustments regarding structural and functional adaptations at the level of all cellular elements and offers a wide range of biotechnological applications; yeasts are especially versatile and, thus, a relevant microbial group in this context [25,27,50].

Further biodegradation, genomic, and transcriptomic studies are planned and should help in elucidating the degradation pathways of lignin-derived aromatic monomers by the strain *R. colostri* DBVPG 10655.

## 5. Conclusions

The data reported in this study demonstrate the capability of the cold-adapted yeast strain *R. colostri* DBVPG 10655 to fully degrade a range of lignin-derived aromatic monomers (5 mM CA, 5 mM HBA, 5 mM FA). Each of these compounds was not only utilized as sole carbon source; full biodegradation occurred also in a mixture of the compounds. In addition, 5 mM VA was degraded in the presence of CA, HBA, and FA (each 5 mM); however, VA could not be utilized as the sole carbon source. Lignin derivate biodegradation in mixtures of multiple compounds (CA, HBA, FA, VA) was observed over a broad temperature range (1–25 °C), which is of ecological and biotechnological relevance. This is the first description of the degradation of lignin-derived aromatic monomers by a yeast strain at low temperatures. The degradation of lignin model compounds has been extensively studied in bacteria and filamentous fungi, while information on the potential of yeasts is limited. Our study adds knowledge on the utilization of lignin-derived aromatic compounds by yeasts in general and under cold-temperature conditions in particular.

## Data Availability

Not applicable.

## References

[1] Brink D.P., Ravi K., Liden G., Gorwa-Grauslund M.F. (2019). Mapping the diversity of microbial lignin catabolism: Experiences from the eLignin database. Appl. Microbiol. Biotechnol..

[2] Bugg T.D.H., Ahmad M., Hardiman E.M., Singh R. (2011). The emerging role for bacteria in lignin degradation and bio-product formation. Curr. Opin. Biotechnol..

[3] Hainal A.R., Capraru A.M., Volf I., Popa V.I. (2012). Lignin as a carbon source for the cultivation of some *Rhodotorula* species. Cellul. Chem. Technol..

[4] Ganewatta M.S., Lokupitiya H.N., Tang C.B. (2019). Lignin biopolymers in the age of controlled polymerization. Polymers.

[5] Lubbers R.J.M., Dilokpimol A., Visser J., Makela M.R., Hilden K.S., de Vries R.P. (2019). A comparison between the homocyclic aromatic metabolic pathways from plant-derived compounds by bacteria and fungi. Biotechnol. Adv..

[6] Tian J.H., Pourcher A.M., Bouchez T., Gelhaye E., Peu P. (2014). Occurrence of lignin degradation genotypes and phenotypes among prokaryotes. Appl. Microbiol. Biotechnol..

[7] Palazzolo M.A., Kurina-Sanz M. (2016). Microbial utilization of lignin: Available biotechnologies for its degradation and valorization. World J. Microbiol. Biotechnol..

[8] Becker J., Wittmann C. (2019). A field of dreams: Lignin valorization into chemicals, materials, fuels, and health-care products. Biotechnol. Adv..

[9] Sampaio J.P. (1995). Utilization of low-molecular weight lignin-related aromatic compounds for the selective isolation of yeasts: *Rhodotorula vanillica*, a new basidiomycetous yeast species. Syst. Appl. Microbiol..

[10] Ravi K., Garcia-Hidalgo J., Nobel M., Gorwa-Grauslund M.F., Liden G. (2018). Biological conversion of aromatic monolignol compounds by a *Pseudomonas* isolate from sediments of the Baltic Sea. AMB Express.

[11] Margesin R., Volgger G., Wagner A.O., Zhang D.C., Poyntner C. (2021). Biodegradation of lignin monomers and bioconversion of ferulic acid to vanillic acid by *Paraburkholderia aromaticivorans* AR20-38 isolated from Alpine forest soil. Appl. Microbiol. Biotechnol..

[12] Botha A. (2011). The importance and ecology of yeasts in soil. Soil Biol. Biochem..

[13] Sampaio J.P., Vanuden N. (1991). *Rhodotorula ferulica* sp. nov, a yeast that degrades ferulic acid and other phenolic compounds. Syst. Appl. Microbiol..

[14] Huang Z.X., Dostal L., Rosazza J.P.N. (1993). Mechanisms of ferulic acid conversions to vanillic acid and guaiacol by *Rhodotorula rubra*. J. Biol. Chem..

[15] Middelhoven W.J. (1993). Catabolism of benzene compounds by ascomycetous and basidiomycetous yeasts and yeast-like fungi—A literature review and an experimental approach. Antonie Leeuwenhoek.

[16] Rahouti M., Steiman R., Seigle-Murandi F., Christov L.P. (1999). Growth of 1044 strains and species of fungi on 7 phenolic lignin model compounds. Chemosphere.

[17] Sampaio J.P. (1999). Utilization of low molecular weight aromatic compounds by heterobasidiomycetous yeasts: Taxonomic implications. Can. J. Microbiol..

[18] Alvarez-Rodriguez M.L., Belloch C., Villa M., Uruburu F., Larriba G., Coque J.J.R. (2003). Degradation of vanillic acid and production of guaiacol by microorganisms isolated from cork samples. FEMS Microbiol. Lett..

[19] Jarboui R., Baati H., Fetoui F., Gargouri A., Gharsallah N., Ammar E. (2012). Yeast performance in wastewater treatment: Case study of *Rhodotorula mucilaginosa*. Environ. Technol..

[20] Max B., Tugores F., Cortes-Dieguez S., Dominguez J.M. (2012). Bioprocess design for the microbial production of natural phenolic compounds by *Debaryomyces hansenii*. Appl. Biochem. Biotechnol..

[21] Bettio G., Zardo L.C., Rosa C.A., Ayub M.A.Z. (2021). Bioconversion of ferulic acid into aroma compounds by newly isolated yeast strains of the Latin American biodiversity. Biotechnol. Prog..

[22] Guiraud P., Steiman R., Seiglemurandi F., Benoitguyod J.L. (1992). Metabolism of vanillic acid by micromycetes. World J. Microbiol. Biotechnol..

[23] Berger T., Poyntner C., Margesin R. (2021). Culturable bacteria from an Alpine coniferous forest site: Biodegradation potential of organic polymers and pollutants. Folia Microbiologica.

[24] Poyntner C., Kutzner A., Margesin R. (2021). Biodegradation potential and putative catabolic genes of culturable bacteria from an Alpine deciduous forest site. Microorganisms.

[25] Buzzini P., Margesin R. (2014). Cold-Adapted Yeasts. Biodiversity, Adaptation Strategies and Biotechnological Significance.

[26] Margesin R., Collins T. (2019). Microbial ecology of the cryosphere (glacial and permafrost habitats): Current knowledge. Appl. Microbiol. Biotechnol..

[27] Collins T., Margesin R. (2019). Psychrophilic lifestyles: Mechanisms of adaptation and biotechnological tools. Appl. Microbiol. Biotechnol..

[28] Franca L., Sannino C., Turchetti B., Buzzini P., Margesin R. (2016). Seasonal and altitudinal changes of culturable bacterial and yeast diversity in Alpine forest soils. Extremophiles.

[29] Wang Q.M., Yurkov A.M., Goker M., Lumbsch H.T., Leavitt S.D., Groenewald M., Theelen B., Liu X.Z., Boekhout T., Bai F.Y. (2015). Phylogenetic classification of yeasts and related taxa within *Pucciniomycotina*. Stud. Mycol..

[30] Wagner A.O., Markt R., Puempel T., Illmer P., Insam H., Ebner C. (2017). Sample preparation, preservation, and storage for volatile fatty acid quantification in biogas plants. Eng. Life Sci..

[31] Turchetti B., Selbmann L., Gunde-Cimerman N., Buzzini P., Sampaio J.P., Zalar P. (2018). *Cystobasidium alpinum* sp. nov. and *Rhodosporidiobolus oreadorum* sp. nov. from European cold environments and Arctic region. Life.

[32] Daskaya-Dikmen C., Karbancioglu-Guler F., Ozcelik B. (2018). Cold active pectinase, amylase and protease production by yeast isolates obtained from environmental samples. Extremophiles.

[33] Glushakova A.M., Kachalkin A.V. (2017). Endophytic yeasts in *Malus domestica* and *Pyrus communis* fruits under anthropogenic impact. Microbiology.

[34] Ramirez-Castrillon M., Usman L.M., Silva-Bedoya L.M., Osorio-Cadavid E. (2019). Dominant yeasts associated to mango (*Mangifera indica*) and rose apple (*Syzygium malaccense*) fruit pulps investigated by culture-based methods. An. Acad. Bras. Cienc..

[35] Lorenzini M., Zapparoli G. (2019). Yeast-like fungi and yeasts in withered grape carposphere: Characterization of *Aureobasidium pullulans* population and species diversity. Int. J. Food Microbiol..

[36] Kaewkrajay C., Putchakarn S., Limtong S. (2021). Cultivable yeasts associated with marine sponges in the Gulf of Thailand, South China Sea. Antonie Leeuwenhoek.

[37] Kaewkrajay C., Chanmethakul T., Limtong S. (2020). Assessment of diversity of culturable marine yeasts associated with corals and zoanthids in the Gulf of Thailand, South China Sea. Microorganisms.

[38] Masiulionis V.E., Pagnocca F.C. (2017). *Rhodosporidiobolus geoffroeae* sp. nov., a basidiomycetous yeast isolated from the waste deposit of the attine ant *Acromyrmex lundii*. Int. J. Syst. Evol. Microbiol..

[39] Ravi K., Garcia-Hidalgo J., Gorwa-Grauslund M.F., Liden G. (2017). Conversion of lignin model compounds by *Pseudomonas putida* KT2440 and isolates from compost. Appl. Microbiol. Biotechnol..

[40] Lubbers R.J.M., Dilokpimol A., Nousiainen P.A., Visser J., Bruijnincx P.C.A., de Vries R.P., Cioc R.C. (2021). Vanillic acid and methoxyhydroquinone production from guaiacyl units and related aromatic compounds using *Aspergillus niger* cell factories. Microb. Cell Fact..

[41] Shen Y., Li H.X., Wang X.N., Zhang X.R., Hou J., Wang L.F., Gao N., Bao X.M. (2014). High vanillin tolerance of an evolved *Saccharomyces cerevisiae* strain owing to its enhanced vanillin reduction and antioxidative capacity. J. Ind. Microbiol. Biotechnol..

[42] Falconnier B., Lapierre C., Lesagemeessen L., Yonnet G., Brunerie P., Colonnaceccaldi B., Corrieu G., Asther M. (1994). Vanillin as a product of ferulic acid biotransformation by the white-rot fungus *Pycnoporus cinnabarinus* I-937: Identification of metabolic pathways. J. Biotechnol..

[43] Ronnander J., Ljunggren J., Hedenstrom E., Wright S.A.I. (2018). Biotransformation of vanillin into vanillyl alcohol by a novel strain of *Cystobasidium laryngis* isolated from decaying wood. AMB Express.

[44] Upadhyay P., Singh N.K., Tupe R., Odenath A., Lali A. (2020). Biotransformation of corn bran derived ferulic acid to vanillic acid using engineered *Pseudomonas putida* KT2440. Prep. Biochem. Biotechnol..

[45] Wang J.X., Liang J.D., Gao S. (2018). Biodegradation of lignin monomers vanillic, p-coumaric, and syringic acid by the bacterial strain, *Sphingobacterium* sp. HY-H. Curr. Microbiol..

[46] Gu H.Q., Zhu Y.Y., Peng Y.F., Liang X.J., Liu X.G., Shao L.Z., Xu Y.Y., Xu Z.H., Liu R., Li J. (2019). Physiological mechanism of improved tolerance of *Saccharomyces cerevisiae* to lignin-derived phenolic acids in lignocellulosic ethanol fermentation by short-term adaptation. Biotechnol. Biofuels.

[47] Liu Z.J., Fels M., Dragone G., Mussatto S.I. (2021). Effects of inhibitory compounds derived from lignocellulosic biomass on the growth of the wild-type and evolved oleaginous yeast *Rhodosporidium toruloides*. Ind. Crop. Prod..

[48] Margesin R., Gander S., Zacke G., Gounot A.M., Schinner F. (2003). Hydrocarbon degradation and enzyme activities of cold-adapted bacteria and yeasts. Extremophiles.

[49] Prem E.M., Mutschlechner M., Stres B., Illmer P., Wagner A.O. (2021). Lignin intermediates lead to phenyl acid formation and microbial community shifts in meso- and thermophilic batch reactors. Biotechnol. Biofuels.

[50] Buzzini P., Turchetti B., Yurkov A. (2018). Extremophilic yeasts: The toughest yeasts around?. Yeast.

